# Tibetan Herbal Pain-Relieving Plaster for Chronic Musculoskeletal Pain Among Cancer Survivors: Study Protocol of a Randomized, Double-Blind, Placebo-Controlled Trial

**DOI:** 10.3389/fphar.2022.878371

**Published:** 2022-05-04

**Authors:** Mingxiao Yang, Raymond E. Baser, Susan Q. Li, Yen-Nien Hou, Kamyar Chong, Yi Lily Zhang, Irfan Hoque, Ting Bao, Jun J. Mao

**Affiliations:** ^1^ Department of Medicine, Integrative Medicine Service, Memorial Sloan Kettering, New York, NY, United States; ^2^ Department of Epidemiology and Biostatistics, Memorial Sloan Kettering, New York, NY, United States; ^3^ Investigational Drug Service, Memorial Sloan Kettering Cancer Center, New York, NY, United States

**Keywords:** Tibetan herbal patch, efficacy, safety, study design, randomized clinical trial, cancer survivor, chronic pain, patient reported outcome (PRO)

## Abstract

Chronic pain is common and debilitating in cancer survivors. Tibetan herbal pain-relieving plaster is used as an external analgesic to treat musculoskeletal pain in China; however, its safety and efficacy have not been evaluated via clinical trials in cancer survivors. We designed this Phase II randomized, double-blind, placebo-controlled trial (ClinicalTrials.gov Identifier: NCT04916249) to assess the efficacy and safety of the pain-relieving plaster for temporary pain relief among cancer survivors with chronic musculoskeletal pain. Under ethical approval from the Institutional Review Board at the Memorial Sloan Kettering Cancer Center, we will enroll eligible cancer survivors who have a clinical diagnosis of moderate to severe chronic musculoskeletal pain in this study. We use a central randomization system to allocate the eligible participants to either the treatment or the control group in a 1:1 ratio, with stratification by baseline opioid use. We will instruct the participants to apply the herbal patch (Tibetree Pain-Relieving Plaster, Tibet Cheezheng Tibetan Medicine Co. Ltd., Tibet, China) or placebo patch daily at the focal area with worst pain for 14 consecutive days. Study physician, participant, outcome assessor, and biostatistician are blinded to the group allocation. The primary outcome is pain severity measured by the Brief Pain Inventory on Days 2–7. Secondary outcomes include changes in insomnia, anxiety, depression, fatigue, pressure pain threshold, pain medication use, and global impression of change. We will also monitor the adverse events throughout the study period. Statistical analysis will follow the intention-to-treat principle and linear mixed modeling will be used. With rigorous design and implementation, this randomized, placebo-controlled trial will provide the initial evidence on the efficacy and safety of the pain-relieving plaster for pain relief among cancer survivors with chronic musculoskeletal pain.

## 1 Introduction

In the United States (U.S.), the number of cancer survivors exceeded 16.9 million in 2019, which is projected to reach 22.1 million by 2030 (Miller et al., 2019). One in three cancer survivors suffer from chronic pain that restricts their daily function, at a rate double that of the general population (Jiang et al., 2019). Chronic pain conditions are often poorly addressed and pose a high symptom burden for cancer survivors as it can substantially impact function and quality of life. Current pain control practices commonly involve analgesics such as nonsteroidal anti-inflammatory drugs (NSAIDs) as the first-line therapy, and opioids and co-analgesics in escalated cases (Nersesyan and Slavin 2007; Paice and Ferrell 2011; Thapa et al., 2011; Schmidt-Hansen et al., 2015). However, pain medications are frequently associated with increased risk of cardiovascular, gastrointestinal side effects and incomplete response rate (Pilotto et al., 2003; Gooch et al., 2007; Harirforoosh et al., 2013; Chou et al., 2015; Ungprasert et al., 2015; Wongrakpanich et al., 2018). Long-term use of opioid drugs in cancer patients is particularly concerning due to opioid dependence, addiction, abuse, and overdose (Benyamin et al., 2008; Palos 2008; Baldini et al., 2012). Further, 20% of patients with cancer pain do not respond to standard analgesic and require second line agents or non-pharmacological interventions (Zech et al., 1995). It is estimated that 5.4 million cancer survivors are still living with poorly managed chronic pain despite current pain management strategy (Jiang et al., 2019), which highlights the unmet need of novel therapeutic approaches.

In the Asia-Pacific region and the U.S., Tibetree Pain-Relieving Plaster (PRP) (National Drug Code: 66506-186)–a patented herbal medicine made with 1% Camphor (Cinnamomum camphora (L.) Nees and Eberm) and other herbal species (Table 1)–is used as an over-the-counter external analgesic for the relief of various musculoskeletal pains (Wen et al., 2019). In an NCI-designated cancer center, patients who used PRP reported improvements in pain that has not been satisfactorily addressed by oral analgesics (Liou et al., 2021). Herbs in PRP have antinociceptive and anti-inflammation effects, through decreasing C-fiber afferent spontaneous firing and reducing the release of inflammatory cytokines (Peng et al., 2011; Duan et al., 2013). Our systematic review revealed that PRP was associated with clinical improvements in pain and joint function for musculoskeletal pain among non-cancer populations (Chen et al., 2020; Yang et al., 2021a). Nonetheless, no placebo-controlled randomized trial has been conducted. Additionally, its effect remains unknown in cancer survivors. Therefore, we designed this phase II study to 1) evaluate the preliminary efficacy and safety of two-week PRP treatment versus placebo for immediate pain relief among cancer survivors with chronic musculoskeletal pain, 2) obtain estimates of change in comorbid insomnia, anxiety, depression, and fatigue, quality of life, global impressions of change, and pain medication usage, 3) explore potential clinical and/or biological traits in association with analgesic response to treatment, such as patient expectancy, pressure pain threshold (Diatchenko et al., 2013).

**TABLE 1:** Composition of the Tibetan herbal pain-relieving plaster.

CHN Pinyin	ENG Name	CHN Name	Scientific Name of Plant^b^	Family Name	Medicinal Parts
*Zhangnao*	*Camphor* ^a^	樟脑	*Cinnamomum camphora* (L.) Nees and Eberm	*Lauraceae* Juss	Bark
*Duyiwei*	*Lamiophlomis Herba* ^a^	独一味	*Phlomis rotata* Benth. ex Hook.f	*Lamiaceae* Martinov	Whole herb
*Jianghuang*	*Curcumae Longae Rhizoma* ^a^	姜黄	*Curcuma longa* L	*Zingiberaceae* Martinov	Rhizome

a According to the Pharmacopeia of China 2015.

b Tropicos v3.3.2 (Tropicos.org. Missouri Botanical Garden. 30 March 2022 <https://tropicos.org>).

## 2 Methods and Analysis

### 2.1 Study Design

This is a prospective, single-center, two-arm, randomized, placebo-controlled trial comparing Tibetree PRP with a placebo control plaster for pain relief in cancer survivors. We plan to enroll 66 eligible patients who completed active cancer treatment and have no evidence of disease, with moderate-to-severe musculoskeletal pain for at least 3 months, from the Memorial Sloan Kettering (MSK). We use a central randomization system to allocate the participants to the experimental or control group in a 1:1 ratio. In the experimental group, patients will be instructed by a study physician/investigator to topically administrate PRP at the worst pain area daily for 14 days. In the control group, patients will be instructed by the same clinician to use the placebo patch at the same frequency for the same time course. The placebo patch mimics the color, smell, dose form, and package of the true patch. The primary outcome is pain severity measured by the brief pain inventory (BPI) at days 2–7. Secondary outcomes include pain severity measured at other time points, pain interference, fatigue, insomnia, anxiety and depression, global impression of change, treatment expectancy, and pressure pain threshold (Table 2).

**TABLE 2:** Study Schedule

TIMEPOINT		Study Period														
	Enroll	Post-allocation													Close-out
	D0	D1	D2	D3	D4	D5	D6	D7	D8	D9	D10	D11	D12	D13	D14
ENROLMENT															
Eligibility screen	X														
Informed consent	X														
Allocation	X														
INTERVENTIONS															
True Patch			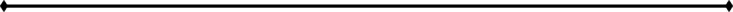												
Placebo Patch			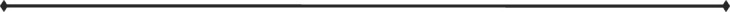												
ASSESSMENTS															
Brief Pain Inventory ^1^	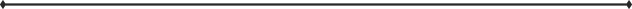														
Demographics and clinical		X													
Pain History	X														
Brief Fatigue Inventory		X						X							X
Hospital Anxiety and Depression Scale		X						X							X
PROMIS-10 Global Health		X						X							X
Patients’ Global Impression of Change			X					X							X
Study Allocation Belief															X
MAO Expectancy of Treatment Effects and of Side Effects of Treatment		X													
Pressure Pain Threshold test ^2^		X													X
Self-reported adherence questionnaire ^1^			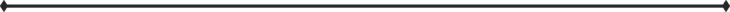												
Pain Medication Diary			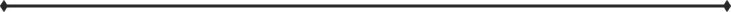												

Key: 1. At Baseline/Day 1, Day 7 and Day 14, patients will complete the entire Brief Pain Inventory. On other days, patients will only complete 4 questions in pain severity subscale of the Brief Pain Inventory. 2. Pressure Pain Threshold will be performed four times during the study period. Three times during baseline visit. First before pain relieving plaster/placebo is applied using FPX Wagner algometer; Second before pain relieving plaster/placebo is applied using MAST system; Third time is 45 ± 15 min after pain relieving plaster/placebo is applied using FPX Wagner algometer. The 4th test will be performed at Day 7 using FPX Wagner algometer.

### 2.2 Participants

#### 2.2.1 Eligibility Criteria

We will enroll cancer survivors with chronic musculoskeletal pain from MSK and the local areas. The following criteria are used to determine the eligibility of a participant.

• Inclusion Criteria: 1) Age ≥18 years or older; 2) A diagnosis of cancer with no restrictions placed on type of cancer or stage. Eligibility criteria will not be restricted to MSK confirmed biopsy/diagnosis; 3) Completed active treatment (surgery, chemotherapy, and/or radiotherapy) at least 1 month prior to study initiation (patients on continued hormone treatment or maintenance targeted therapies will not be excluded); 4) Patients currently have no evidence of disease; 5) Ambulatory (Karnofsky functional score of ≥60); 6) Having a focused location of regional musculoskeletal pain (e.g. joints, extremities, back, neck) that can be covered by one patch of PRP; 7) Worst pain score (on a 0-10 numeric pain rating scale) ≥ 5 in the preceding week; and 8) Pain for at least 3 months and at least 15 days with pain in the preceding 30 days; and 9) Willingness to adhere to and understanding of all study-related procedures, including randomization to one of the two possible treatment groups; (10) Able to understand informed consent and provide signed informed consent form.
• Exclusion Criteria: 1) Patients with non-musculoskeletal pain syndromes (headache, facial pain, chest pain, visceral abdominal pain) will be excluded if these are the sole source of pain but can be present as co-morbid conditions as long as the patient has a primary musculoskeletal pain condition defined as above; 2) Patients have generalized musculoskeletal pain such as fibromyalgia; 3) Use of corticosteroid drugs by any route of administration within 30 days; 4) Patients with significant self-reported skin disorders; 5) Patients with open wounds, infections, skin trauma at skin overlying area of pain; 6) Patients with documented history of skin sensitivity to adhesive or allergic reaction to other patches or topical analgesics; 7) Patients with documented skin allergic reaction to plants or herbs; 8) Patients who are in active treatment (chemotherapy, surgery, radiotherapy); 9) Plan to change or initiate other pain medications or interventions (e.g. physical therapy, acupuncture, injection). Patients may remain on their current pain regimen.

#### 2.2.2 Recruitment Strategy

Our primary recruitment approach will use a population-based strategy via sending recruitment letters to potential participants. Potential candidate will be identified through Dataline querying at MSK by an analyst. Our clinical research coordinators (CRC) (KC) will reach out to potential participants identified or referred to the study coordinator by protocol investigators or other MSK healthcare providers. We will also enroll self-referred participants. Information about the protocol will be provided in lay language on MSK’s web site and on clinicaltrials.gov. Additionally, printed materials will be posted in clinic areas (e.g., MSK’s solid tumor clinics, cancer survivorship clinic, and integrative medicine clinic).

#### 2.2.3 Screening and Research Participant Registration

A CRC will screen potential interested and eligible patients, and schedule screening and consenting appointments. A study investigator will confirm each patient’s eligibility and complete a protocol-specific Eligibility Checklist to ensure that all institutional requirements necessary to enroll the participant to the study have been completed. A CRC specially trained for the study will obtain informed consent. After establishing eligibility and consenting, the study staff will register participants through the Clinical Trials Management System (CTMS).

### 2.3 Randomization and Allocation Concealment

Participants will be randomized in a 1:1 ratio to the PRP or placebo plaster groups using MSK’s Clinical Research Database (CRDB), a secure computer system that ensures full allocation concealment. Permuted block randomization methods with stratification by baseline opioid use (yes/no) will be utilized. Participants’ treatment assignments can be viewed in the CRDB only by the hospital pharmacists who are dispensing the study medication.

### 2.4 Blinding

The CRCs, patients, clinicians, PI, co-investigators, biostatisticians will be blinded to the group allocation. The MSK research pharmacy will maintain the randomization and distribution list of the PRP and placebo medications. Pharmacist who dispenses the patches will have no contact with patients and a study clinician will pick up the plasters from the pharmacy and distribute them to patients on their Day 1 visit.

### 2.5 Interventional Methods

#### 2.5.1 Intervention Groups

• PRP: Each participant will receive 14 plaster packages in a single box in the PRP group from our study clinician (MY). Each package includes a light brown plaster (1.2 g/patch) and a small white plastic bag of dark yellow herbal liquid (1% camphor [Cinnamomum camphora (L.) Nees and Eberm], 2.5 ml/bag). The plaster is composed of a paper-based hypoallergic medical adhesive (122 mm × 145 cm) and at its center a single layer (thickness 0.8 mm) of sealed cotton pad (50 mm × 70 mm) with herbal powders inside. The herbal components of PRP are detailed in Table 1.
• Placebo: The placebo plaster lacks the active ingredients but are otherwise identical to the true patch in appearance. The placebo pouch contains liquid made up of purified water, sodium methyl para-hydroxybenzoate, sodium propyl 4-hydroxybenzoate, sodium dodecyl sulfate, and tartrazine. The placebo herbal powders in the plaster are composed of plant fiber, light yellow dye, yellow dye, egg yolk powder, cocoa powder, pumpkin powder, and active charcoal. Tibet Cheezheng Tibetan Medicine Co. Ltd. (Lingzhi city, Tibet, China) manufactured both the real and the placebo patch (lot number: 202110301/202110402; production date: March 2021; expiry date: November 2023).

#### 2.5.2 Patch Use Instructions

On Day 1, the study clinician will help the patient identify a focused body area where the patient reports worse pain. This will be the area for the patch to be applied. The study clinician will demonstrate the patient how to appropriately apply the patch. To use, we advise each patient to adopt the following procedures: 1) clean the skin of the affected area, 2) remove the plastic film from plaster, 3) squeeze the diluents contained in the plastic bag onto the middle of the pad, and 4) apply the plaster on the affected area. We remind all patient that a patch should not be worn for more than 6 h to avoid any potential skin irritation or unknown side effects. The fidelity of treatment is ensured by drug use compliance recording form which is automatically sent to patient daily through the REDCap system. In the following days, patients are required to apply only one patch daily at the fixed pain area. At the end of the study, we also require all patients to return the drug box and any unused patches.

### 2.6 Outcomes

#### 2.6.1 Primary Outcome

The primary outcome is pain severity measured with the Brief Pain Inventory (BPI) at days 2–7. The BPI is a 11-item pain instrument validated to quantify pain severity (4 items) and pain interference (7 items). It is one of the most widely used instruments to measure pain in patients and has been demonstrated to be a reliable, valid, and responsive measure (Cleeland and Ryan 1994). We define treatment response as a 30% or greater reduction in the pre-post intervention pain severity score (Farrar et al., 2001). BPI should be completed prior to removing pain relieving plaster at the end of each day.

#### 2.6.2 Secondary Outcomes

• Pain severity and interference measured by the BPI at day 14.
• Patients’ Global Impression of Change (PGIC) is a one item survey that will be used to define a clinically important change in pain from the patient’s perspective (Sloan et al., 2002). Patients will be asked “Compared to when the study began, how would you describe your pain now? I am: very much worse, much worse, a little worse, the same, a little improved, much improved, very much improved.” The PGIC can be used as an anchor to derive anchor-based minimally important differences (MIDs) for pain measures like the BPI.
• Brief Fatigue Inventory (BFI) will be used to determine the effect of treatments on fatigue. This 9-item instrument was designed to assess one construct of fatigue severity in cancer and non-cancer populations. Three items ask patients to rate the severity of their fatigue at its “worst,” “usual,” and “now” during normal waking hours, with 0 being “no fatigue” and 10 being “fatigue as bad as you can imagine.” Six items assess the amount that fatigue has interfered with different aspects of the patient’s life during the past 24 h. The interference items are measured on a 0–10 scale, with 0 being “does not interfere” and 10 being “completely interferes” (Sloan et al., 2002). A composite fatigue severity score can be found by averaging the nine item scores. The score of the scale was found to be reliable and valid in multiple languages and diverse populations (Sloan et al., 2002; Lin et al., 2006).
• Insomnia Severity Index (ISI) will be used to measure subjective insomnia severity. The ISI has seven items rated on a 5-point Likert response scale (e.g., 0 = no problem; 4 = very severe problem), yielding a total score ranging from 0 to 28 with higher scores representing more severe insomnia symptoms. The usual recall period is the “last month”; however, we will use a modified recall period of “the past week” to match our assessment schedule. The ISI authors suggest the following guidelines for interpreting the ISI total score: < 8, no clinically significant insomnia; 8-14, subthreshold insomnia; 15-21, clinical insomnia (moderate severity); >21, clinical insomnia (severe).(Bastien et al., 2001). The ISI has demonstrated internal consistency, reliability, construct validity, specificity and sensitivity in a representative sample of 1670 cancer patients.(Savard et al., 2005). The ISI has established minimally important change values to ensure that the change is not only statistically, but also clinically, meaningful to patients.(Morin et al., 2011). A reduction of eight points has been deemed to be clinically significant improvement. (Morin et al., 2011).
• Hospital Anxiety and Depression Scale (HADS) will be used to explore the effect of treatments on psychological distress. HADS is a 14-item scale with seven items measuring depression and seven items measuring anxiety. Each item is answered by the patient on a four-point (0–3) response category so possible scores range from 0-21 for anxiety and depression, with higher scores indicating higher symptomatology. Established cutoffs are: 0–7 not significant; 8–10 subclinical; and 11–21 clinically significant depression/anxiety.(Zigmond and Snaith 1983). Factor analysis showed two distinct but correlated factors of anxiety and depression.(Moorey et al., 1991). The scale scores have been shown to be both reliable and valid.(Smith et al., 2002).
• Patient Reported Outcomes Measurement Information System (PROMIS®) Scale v1.2 - Global Health is a brief instrument composed of 10 items that demonstrates adequate reliability and validity (Hays et al., 2009; Revicki et al., 2009) as a measure of health related QOL in general and clinical populations.(Rothrock et al., 2010; Barile et al., 2013). Patients are asked to respond to questions 1-8 and 10 on a scale of 1–5. Question nine is on a 0–10 scale (average pain rating). The measure yields two scores, Physical Health and Mental Health, that will be used as secondary outcomes to evaluate the effect of Tibetree PRP on QOL.(Hays et al., 2009). These scores will be calculated using item-level calibrations based on item response theory (IRT) scaling and then transformed to T-Scores, which are standardized such that 50 represents the mean for the US general population, and the standard deviation around that mean is 10 points.(Hays et al., 2009). Higher scores indicate better Physical and Mental Health.
• MAO Expectancy of Treatment Effects (METE) has four items originally developed as the Acupuncture Expectancy Scale (AES) by Mao et al.(Mao et al., 2007) It has demonstrated reliability (Cronbach’s α of 0.82) and validity and is positively correlated with patient self-reported efficacy and satisfaction.(Mao et al., 2007). The score ranges from 4 to 20, with higher scores indicating greater expectancy.
• Mao Expectancy of Side Effects of Treatment (MESET) measures the expectations of side effects and therapeutic effects of the study intervention.(Mao et al., 2014). The score ranges from 4 to 20, with higher scores indicating greater expectancy for side effects. We will use the METE and MESET to explore whether expectancy predicts treatment outcomes and may impact the observed differences between groups.
• Pressure Pain Threshold: This is a study measure that a study investigator will administer. Pressure algometry is a reliable measure of pressure pain threshold. Two types of algometers will be used in this trial:
  (1) the FPX Wagner algometer: We will use a digital pressure algometer, FPX (Wagner instrument, Greenwich, United States), to apply pressure at the site of pain (the tender points of tissue where the patient experiences the worst pain) and two pain-neutral areas (thumbnail bed and trapezius) to derive the pressure pain threshold (the first pressure that is perceived as painful) shown to be predictive of analgesic effects (Harte et al., 2013a; Wasserman et al., 2015). The clinical researcher will apply pressure at a 0.5 kgf/cm^2^/sec increment at the tissue located at or surrounding the tested site with the tip of the algometer. The test will be terminated when participants reach their pain threshold or a pressure of 10 kgf/cm^2^. The test will be repeated three times per site, and we will take the mean threshold for analysis, measured as kgf/cm^2^. The test will be conducted three times during the study: before patch application at baseline; 45 min after patch application at baseline and at the primary endpoint (Day 7).
  (2) the MAST system: We will use the MAST System (Arbor Medical Innovations, Ann Arbor, MI) (Harte et al., 2013b; Schrepf et al., 2015; Wasserman et al., 2015; Treister et al., 2018; Harte et al., 2019) to apply computer-controlled pressure at the thumbnail bed to derive the PPT as well as suprathreshold measures of pain sensitivity (e.g., pain tolerance) shown to be predictive of analgesic effects.(Harte et al., 2013a; Wasserman et al., 2015). The system delivers ascending and random patterns of discrete pressures (5 s duration; 4 kgf/cm^2^/s ramp rate) at 20 s intervals, beginning at 0.25 kgf/cm^2^ and increasing in 0.25–0.50 kgf/cm^2^ steps. Pain intensity will be rated after each stimulus on a 0–100 Numerical Rating Scale. The test will be terminated when participants reach their tolerance or 10 kgf/cm^2^. PPT test with the MAST system will be conducted once before patch application at baseline only. The validity of testing pressure pain threshold with the MAST system has been demonstrated extensively (Petzke et al., 2001; Gracely et al., 2002; Giesecke et al., 2003; Petzke et al., 2003; Petzke et al., 2005; Harris et al., 2006; Geisser et al., 2008; Harris et al., 2008; Harris et al., 2009; As-Sanie et al., 2013; Henry et al., 2014; Clauw 2015). We will use this measure to explore whether pressure pain threshold predicts treatment outcomes and impacts the observed differences between groups. We will also incorporate the pressure pain threshold into the clinical outcomes to provide more objective, less biased quantification of pain and more accurate assessment of the analgesic effect of the herbal patches.
• Study Allocation Belief: This is a patient-reported outcome that asks patient to guess which treatment they have received during the study to assess the adequacy of blinding.
• Pain Medication Diary: Name of pain medication, the dosage/strength, number of days patients took the medication and how many pills were taken were collected in the Pain Medication Diary. A pain medication usage score was calculated according to the method developed by Robinson-Papp et al. (Robinson-Papp et al., 2015).
• Adverse events: We will document all related adverse events and report any related serious adverse event promptly to IRB.

### 2.7 Sample Size Estimation

The main objective of this study is to provide preliminary data on the effect of PRP in reducing musculoskeletal pain compared to placebo plaster. We conservatively anticipate lost to follow up to be at most 10% by day 7. To ensure adequate power to compare arms on our secondary symptom endpoints, we will enroll 66 patients to obtain at least 60 patients evaluable for these secondary symptom endpoints. Our sample size will provide sufficient statistical power to detect clinically relevant effect sizes for our primary pain outcome between PRP vs. placebo plaster (primary objective). We present our power considerations for an ANCOVA regression model (e.g., predicting Day 7 pain score controlling for baseline pain score), which we will apply to our primary pain endpoint as well as to the secondary symptom endpoints. However, our analysis method for our primary endpoint (linear mixed model) will be able to detect a slightly smaller effect size than presented here. For our sample size/power considerations, we calculated the smallest standardized effect size we will be able to detect with 0.80 power, given a sample size of 60. With 30 participants in each of the two arms, we will have power of 0.80 to detect an effect size of 0.74 (standardized mean difference, Cohen’s d) between the two arms at Day 7, using a two-sided test with alpha of 0.05 in a regression model controlling for baseline score. This 0.74 effect size is considered a moderate-to-large effect size by convention. Given the standard deviation of the BPI pain severity subscale typically ranges between 2 and 3, this effect size translates to a mean difference between the arms of around 1.5 to 2.25 points on the 0–10 BPI scale. A 1-point difference in BPI severity is clinically important (Dworkin et al., 2008). In addition, this criteria is also used in a recent high-impact publication comparing opioid vs. nonopioid medication for chronic low back pain (Krebs et al., 2018). Even if we find no statistically significant difference between the arms on the primary endpoint, we will estimate and report the observed effect size and 95% confidence interval to assist in planning potential larger trials.

### 2.8 Statistical Analysis Plan

We describe the analysis for the primary objective below using the intention-to-treat (ITT) principle. We will also perform a per-protocol sensitivity analysis among treatment and assessment completers, but study conclusions will be based upon the ITT analysis results.

#### 2.8.1 Populations for Analyses

All randomized patients will have a baseline pain assessment, and those with at least one post-baseline BPI assessment on or before Day 7 will be included in the analysis of the primary objective using the modified ITT principle (i.e. participants will be analyzed according to the treatment group to which they will be randomly allocated regardless of treatment adherence status). Secondary outcome such as insomnia, anxiety, depression, fatigue, and quality of life are measured at baseline, Day 7, and Day 14. The analysis population for these endpoints will include patients who complete the baseline and one or both of the Day 7 and Day 14 assessments. Pressure pain threshold is measured at baseline before patch application, baseline 45 min after patch application and Day 7. The analysis population for this endpoint will include patients who complete the test at baseline before patch application and one or both of the other two timepoints. Additionally, all patients who apply at least 1 of the study plasters will be included in the analysis of safety.

#### 2.8.2 Analysis Plan for Primary Objective

We will plot the outcome measure trajectories by randomization arm over time and summarize each outcome measure at each assessment time by treatment arm using descriptive statistics. We expect patients’ pain levels to decrease upon first application of the PRP and then stay at a similarly decreased level with continued use over the study period. Accordingly, we will test for differences between the two study arms in post-Baseline BPI severity using a linear mixed model (LMM) predicting post-Baseline BPI severity as a function of treatment arm, controlling for Baseline BPI severity, assessment time, and randomization strata (baseline opioid use, yes/no). The test of differences between randomization arms with respect to the outcome will be based on the coefficient for treatment arm in this LMM. For our test of differences between the arms in the primary endpoint (Day 2–7 BPI severity), the LMM will have BPI severity assessments through Day 7. We will fit a similar model with BPI assessments through Day 14, but the Day 7 model will be our primary test of efficacy.

To enhance patient-centered data interpretation and decision-making, we will also perform responder analyses by considering those who experienced 30% or greater reduction in BPI severity as responders at the primary end point/Day 7 and at the end of treatment/Day 14.(Farrar et al., 2000; Farrar et al., 2001; Farrar et al., 2010). We will compare the proportion of responders in PRP and placebo plaster groups at both time points using descriptive cross-tabulations and logistic regression adjusting for the baseline outcomes and randomization strata.

#### 2.8.3 Analysis Plan for Secondary Objectives

The number and percent of adverse events within each treatment arm will be reported by grade. We will compare changes between the arms in insomnia, anxiety, depression, fatigue, quality of life, and pain medication usage scores using linear regressions predicting these endpoint scores at Day 7 and Day 14 (separate models) as a function of treatment arm, controlling for baseline score and randomization strata. This is equivalent to an ANCOVA model. The same analysis will be performed for PPT for the baseline (45 min after patch application) and Day 7 assessments, controlling for the pre-patch baseline assessment. We will summarize responses to the PGIC at days 2, 7, and 14 using frequencies and percentages by arm, and test for differences between arms using chi-square tests. To obtain estimates of the correlation of treatment expectancy and treatment response, we will fit a LMM similar to that specified for the primary objective, but with the addition of METE score and the interaction between treatment arm and METE score. To evaluate whether PPT predicts treatment response, we will fit a LMM similar to that specified for the primary objective, but with the addition of baseline (pre-patch) PPT score and the interaction between treatment arm and PPT baseline (pre-patch) score. Pain response variations in associated with other variables, such as sex, race/ethnicity, or age, may be addressed in post hoc analyses.

## 3 Discussion

The current opioid epidemic has presented enormous challenges to the management of pain among cancer survivors (Vitzthum et al., 2020). Complementary and integrative medicine is widely used for the relief of pain among global populations (Chen and Michalsen 2017). Tibetan PRP as an OTC topical analgesic is used by patients with musculoskeletal disorders and chronic pain in China and more recently in the U.S. We designed this phase II trial to inform the preliminary efficacy and safety of PRP among cancer survivors experience chronic pain.

In real-world practice, PRP used as a topical analgesic confers pain relief and clinical benefits that are comparable to oral analgesics to cancer patients (Liou et al., 2021). Clinical evidence demonstrates that among patients with osteoarthritis (OA), PRP compared to pain medications such as NSAIDs, glucosamine, and intraarticular corticosteroid are associated with greater improvements in pain severity, stiffness, and joint function (Chen et al., 2020). However, current studies are limited in the randomization process and the selection of control methods, causing major selection bias and performance bias (Chen et al., 2020; Yang et al., 2021a). Till now, no randomized placebo-controlled trial has been conducted to evaluate the effect of PRP for chronic musculoskeletal pain. The lack of relevant evidence further ponders clinical decision-making process. Therefore, quality evidence is required to inform the application of PRP for pain relief among cancer populations.

Results of our study will contribute to understanding the efficacy and safety of PRP for temporary pain relief among cancer survivors with chronic musculoskeletal pain. Our systematic review of randomized controlled trials showed that PRP compared to analgesic medications such as Diclofenac or NSAIDs may lead to greater improvements in pain outcomes in acute low back pain patients (Yang et al., 2021a). Building on preliminary data, we hypothesized that PRP, within a two-week treatment window, may allow us to detect possible improvements in pain compared with placebo among cancer survivors with chronic musculoskeletal pain. If PRP is found promising for pain management in such a short term, this trial may have important clinical implications, which will provide us with important clinical data to power the design for a long-term definitive trial in the future.

Pain and comorbid symptoms like insomnia and depression may share common neurobiological pathways (Finan and Smith 2013). Recent studies indicate that nonpharmacologic pain interventions like cognitive behavioral therapy for insomnia or acupuncture may improve both pain and comorbid symptoms via regulating these shared biological pathways (Yang et al., 2021b). The present study will also provide insights into the clinical effect of PRP on pain comorbid symptoms that are prevalent in cancer survivors, including emotional distress, fatigue, and insomnia (Pachman et al., 2012). Furthermore, previous studies have linked patient psychosocial characteristics (such as patient expectancy (Smeets et al., 2008)) and pain phenotype (such as pain threshold (Zucker et al., 2017)), to the variations in response to pharmacologic or nonpharmacologic treatments. We will further explore potential phenotypic biomarkers that may predict pain response to treatment.

Although PRP may not impose serious side effects when used as a topical analgesic among chronic pain populations, moderate skin irritations were reported in observational studies, including skin dryness, redness, and blistering in the area where patch is applied (Chen et al., 2020; Yang et al., 2021a). As pharmacosurveillance of potential herb-drug interactions is critical to safety integrative care, this study will also enable us to gain important insights into the safety profile of PRP in cancer survivors. As many survivors are interested in herbal products, this study can potentially guide the establishment of a safe and novel pain control approaches to improve the quality of life for cancer patients.

## 4 Trial Status, Ethics, Data and Safety Monitoring, and Dissemination

We are currently enrolling patients. We designed this study in full compliance with the Declaration of Helsinki and the principles of Good Clinical Practice to maximize the protection of patient rights and health. The Institutional Review Board (IRB) at MSK reviewed and approved all procedures stated in this study protocol version 5.14 (27 May 2021) (Approval number: 20–496). All eligible patients provide written consent or a signed e-consent form prior to the initiation of any study procedures. All patient data will be protected under the MSK CRDB and the REDCap systems. MSK Data and Safety Monitoring Committee oversees the study and initiates review from the enrollment of the first participant, till the protocol has closed to accrual. Results of this study will be disseminated through poster/oral presentations at regional/international conferences and peer-reviewed journals.
